# The effect of different internal fixation materials on Jakob type III lateral humeral condyle fractures in pediatric patients

**DOI:** 10.1186/s13018-025-06547-9

**Published:** 2025-12-02

**Authors:** Xinfeng Wang, Liangfu Xie, Anning Xia, E. Bing, Shaoting Luo, Guibing Fu, Jiansheng Wang

**Affiliations:** 1 Department of Pediatric Orthopedics, Shenzhen Children’s Hospital, No. 7019 Yitian Road, Futian District, Shenzhen, 518000 Guangdong Province People’s Republic of China; 2 Department of Pediatric Orthopedics, Shengjing Hospital of China Medical University, Shenyang, Liaoning, 110004 People’s Republic of China

**Keywords:** Pediatric patients, Lateral condylar fracture, Open reduction, Kirschner wire, Metal hollow screw, Absorbable hollow screw

## Abstract

**Background:**

Humeral lateral condyle fractures are common pediatric elbow injuries, with Jakob type III representing the most severe form. While open reduction and internal fixation is the standard treatment, consensus is lacking on the optimal fixation method. Inadequate fixation can lead to serious complications. The efficacy of absorbable internal fixation materials remains debated. This study aimed to evaluate the outcomes of different internal fixation materials for treating these fractures in children.

**Methods:**

A retrospective review was conducted on 51 children (39 male, 12 female; mean age 4.8 years) with Jakob type III fractures treated surgically between January 2020 and December 2023. Patients were divided into three groups based on fixation: Kirschner wires (Group A), metal hollow screws (Group B), and absorbable hollow screws (Group C). Statistical significance was set at *P* < 0.05.

**Results:**

The average hospitalization was 3.8 ± 0.2 days, median operation time was 60 min, and mean follow-up was 25.2 ± 12.1 months. No significant differences were found among groups regarding gender, age, postoperative plaster fixation time, final carrying angle, elbow varus deformity, lateral condyle protrusion, Mayo elbow score, or satisfaction. However, significant differences existed in hospital stay, operation time, time to implant removal, total hospitalization costs, and material costs. Group B had a significantly longer hospital stay (mean 4.9 days) compared to Groups A and C (3.5 and 3.4 days). Operative time was longest in Group A (mean 60 min vs. 45 and 47 min). Implant removal time was significantly longer in Group B (median 93 days vs. 34 days in Group A). Group B had the highest total hospitalization costs (mean $2623.0), while Group C had the highest material costs (mean $996.5). Group A had the lowest costs in both categories.

**Conclusion:**

Different internal fixation materials achieved comparable therapeutic effects for pediatric Jakob type III lateral condylar fractures. Absorbable screws provided the advantage of eliminating the need for a second surgery for removal.

**Supplementary Information:**

The online version contains supplementary material available at 10.1186/s13018-025-06547-9.

## Introduction

Lateral condylar fractures of the humerus are common types of elbow fractures in children, accounting for 12% to 20% of all distal humeral fractures [1]. The most common age for this fracture is 6 years [2]. There are two commonly used classifications in clinical practice based on the degree of fracture displacement: the Milch classification and the Jakob classification. The Jakob classification also includes the rotation of the fracture fragment, so it is more widely used in clinical practice [3]. Humeral condylar fractures in children are divided into three types according to the Jakob classification: type III are the most serious type of humeral condylar fractures which refer to fractures displaced greater than 4 mm, accompanied by bone rotation, displaced condylar fragments, and soft tissue incarceration at the fracture end [3].

The current treatment strategy for this type of fracture is to adopt the surgical method of open reduction and internal fixation, and a certain period of plaster fixation after the surgery (this period refers to 4–6 weeks after surgery). However, there is no unified consensus on which internal fixation material to use and how to fix it [4–9]. Moreover, if the fracture fragments are not firmly fixed after the fracture reduction, there may be a risk of nonunion. The risk of elbow deformity and necrosis of lateral condyle is very high in the later stage, and it can seriously affect the functional activities of children’s elbow joints [10]. Some complications include valgus deformities, varus deformities, loss of flexion, loss of extension, prominent lateral condyle, fishtail deformity, avascular necrosis, premature epiphyseal closure and neurological deficits [11]. Therefore, stable fixation of bone fragments is crucial to reduce loss of postoperative elbow function.

Previous related studies have shown that Kirschner wire internal fixation materials and metal hollow screw fixation materials can achieve satisfactory fixation effects [12, 13], and in recent years, there have been related literature reporting on the use of absorbable internal fixation materials in the context of orthopedic trauma. Studies have shown that absorbable internal fixation materials have satisfactory fixation effects, do not affect fracture healing, and do not require reoperation to remove the internal fixation. Materials with such advantages can be used for the treatment of humeral external condyle fractures [14]. However, relatively few comparative studies on internal fixation materials for severe humeral condyle fractures in children currently exist. Therefore, we retrospectively analyzed the clinical data of different internal fixation materials for treating patients with Jakob type III humeral condyle fractures in our hospital in recent years to explore the efficacy of different internal fixation materials.

## Material and methods

We conducted a retrospective analysis of the relevant diagnosis and treatment data of 51 children with humeral condylar fractures who underwent surgical treatment in our hospital from January 2020 to December 2023, including 20 cases in group A, 14 cases in group B, and 17 cases in group C. The average age of the sample was 4.8 ± 1.26 years, 76.5% were male (39/51). The mean age was 5.0 ± 1.2 years in group A, 4.5 ± 1.2 years in group B and 4.9 ± 1.3 years in group C, respectively. The inclusion criteria were as follows: 1. Children aged 1–18 years; 2. Preoperative imaging data and intraoperative surgical exploration confirming that the humeral condyle fracture was type III according to the Jakob classification [3]. 3. Time from injury to surgery less than 7 days. 4. Children who underwent open reduction and internal fixation treatment. 5. Children with complete clinical data and follow-up data. The exclusion criteria were: 1. Patients with lateral condyle fracture combined with fractures or joint dislocations in other parts of the body. 2. Patients with lateral condyle fracture combined with important neurovascular injuries. 3. Patients with nontraumatic humeral condylar fractures; 4. Patients with lateral condyle fracture combined with underlying medical conditions (e.g., rheumatoid arthritis, metabolic bone diseases). This study has been approved by the hospital ethics committee.

In this study, three different types of internal fixation implants were mainly used: group A used a titanium alloy Kirschner wire produced by Mindray Technology Co., Ltd., model KZR01YA; group B used CDQ03-P titanium alloy hollow compression screw (half thread type) produced by Dabo Medical Technology Co., Ltd.; in group C, the absorbable hollow screws of B-ASC-35/40 type produced by Bioretec Co., Ltd. were used. The choice of internal fixation materials in different groups was determined by shared decision-making between the surgeons and the patients’ families.

For each patient, we collected demographic data and closely monitored for postoperative complications, which included infection, reoperation, failure of the fixation material, and specific conditions such as:

- Spurring: Defined as an overgrowth of bone on the lateral condyle, resulting in an irregularity of the metaphyseal flare [15].
- Avascular necrosis of the lateral condyle.
- Nonunion.
- Significant nerve and vascular damage postoperatively.

The surgical technique included: 1. The elbow joint was exposed through the Kocher lateral J-shaped approach and dissected to the lateral condyle of the humerus; 2. The joint was irrigated, remove blood clots and tissue debris, the articular surface was reduced anatomically, confirm that the joint has been reset, and a bone holder or towel clamp was used to maintain the reduction; 3. In the Kirschner wire fixation group (Group A), three smooth Kirschner wires were inserted in a crossed configuration: the anterolateral, posterolateral and lateral middle of the lateral humeral condyle fracture block were used as the entry points (all avoiding the epiphyseal plate of the distal humerus and avoiding damage to the growth and development structure). Each Kirschner wire was 45° -60° inclined to the fracture line, and was gradually inserted into the medial direction of the distal humerus metaphysis. The three Kirschner wires were not arranged in parallel, but formed a three-dimensional cross structure: the anterolateral Kirschner wire and the posterolateral Kirschner wire showed a divergence angle of 15°–20° in the coronal plane, and the lateral middle Kirschner wire and the former two showed a cross angle of 10°–15° in the sagittal plane. Finally, the three needles intersected in the fracture block and the metaphyseal region. A smooth Kirschner wire was used to obliquely pass through the fracture block into the medial part of the metaphysis at 45°–60°, and a screw of appropriate size was used to guide the Kirschner wire to twist into the corresponding position. In this process, special attention should be paid to avoiding the epiphyseal plate. Then, the guide Kirschner wire was pulled out (Groups B and C). 4. Before the wound was sutured, Intraoperative fluoroscopy or radiographs were obtained to confirm fracture reduction and implant position, and the elbow was finally immobilized in 90° of flexion with a plaster cast.

All the data were statistically analyzed using SPSS v.26.0 software (IBM Corp). Descriptive statistics were presented as mean ± SD or medians, quartiles, and percentages. The differences between different treatment groups were statistically analyzed using Kruskal–Wallis test, Chi-square test, Mann–Whitney test, Fisher’s exact test, and t-test. All analyses were performed two-sided tests, with a test level of α = 0.05. A *p* value of < 0.05 was considered statistically significant.

## Results

There were 51 patients enrolled in this study, with no patients lost at follow-up. The average hospital stay was 3.8 ± 0.2 days and the average follow-up time was 25.2 ± 12.1 months. There were 29 children (56.9%) with complications after surgery; all of these complications were spurring (including 3 cases with cubitus varus deformity). However, spurring had no significant effect on function or appearance. There were no complications, such as infection, bone nonunion, avascular necrosis of the lateral condyle, neurovascular injury, or failure of internal fixation materials (Table 1).

**Table 1:** Patient’s clinical characteristics

Variable	*N* = 51
Male^①^	39 (76.5%)
Female^①^	12 (23.5%)
Age (year) ^②^	4.8 ± 1.26
Right^①^	21 (41.2%)
Left^①^	30 (58.8%)
Length of hospital stay^a^ (day) ^②^	3.8 ± 0.2
Operative time^b^ (minute) ^③^	50 (45,60)
Time to implant removal^c^(day) ^③^	43 (33,82)
Total hospitalization cost^d^ ($) ^③^	1888.5 (1470.1,2452.4)
Implant cost ($) ^③^	696.6 (298.9,990.9)
Duration of cast immobilization (day) ^③^	33 (30,40)
Cubitus varus^e①^	
Yes	3 (5.9%)
No	48 (94.1%)
Spurring^①^	
Yes	29 (56.9%)
No	22 (43.1%)
Postoperative complication^①^	
No	22 (43.1%)
Yes	29 (56.9%)
Mayo elbow performance score	
Excellent	48 (94.1%)
Good	3 (5.9%)
Fair	0
Poor	0
Implant materials^①^	
Kirschner wire	20 (39.2%)
Hollow screw	14 (27.5%)
Absorbable screw	17 (33.3%)
Follow-up duration (month)^②^	25.2 ± 12.1

^a^(Length of hospital stay): Total time spent on first hospitalization;

^b^(Operative time): time spent on the first surgical treatment;

^c^(Time to implant removal): The time interval between the first fixing of the implant and the last removal of it

^d^(Total hospitalization cost): All treatment costs for first hospitalization (USD)

^e^(Cubitus varus): Measured at the last follow-up

①*n*(%); ②Mean ± SD; ③Median (IQR)

Among the 51 children included in this study, 20 children were in Group A, 14 were in Group B, and 17 were in Group C. 9(45.0%) in group A had fractures on the right side, the median time of postoperative plaster fixation was 33 days, and the median material cost and total hospitalization cost were 291.4$ and 1470.3$, respectively. Ten children developed complications developed spurring after surgery, and 95% of the children had excellent Mayo Elbow Performance Score. The median material cost and total hospitalization cost of Group B were 751.4$ and 2623.0$, respectively, whereas those of Group C were 996.5$ and 2207.0$, respectively. In Group B, two patients (14.3%) had cubitus varus deformity, nine patients (64.3%) had spurring, and 85.7% of the children had very excellent Mayo Elbow Performance Score. No patients in Group C developed cubitus varus, 10 patients (58.8%) had spurring, and all the children had excellent Mayo Elbow Performance Score [16]. No postoperative wound infections occurred in any of the patients (Table 2).

**Table 2:** Clinical characteristics of different internal fixation materials in the treatment group

Variable			Kirschner wire group	Hollow screw group	Absorbable screw group
		*n* = 51	*n* = 20	*n* = 14	*n* = 17
Male^①^		39 (76.5%)	17 (85%)	11 (78.6%)	11 (64.7%)
Female^①^		12 (23.5%)	3(15%)	3 (11.4%)	6 (35.3%)
Age ^②^		4.8 ± 1.26	5 ± 1.2	4.5 ± 1.2	4.9 ± 1.3
Right^①^		21 (41.2%)	9 (45%)	6 (42.9%)	6 (35.3%)
Length of hospital stay^②^		3.8 ± 0.2	3.5 ± 0.2	4.9 ± 0.2	3.4 ± 0.2
Operative time^③^		60 (45,60)	60 (55,95)	45 (40,50)	47 (30,75)
Removal internal fixation^③^		60 (33,82)	34 (31,41)	93 (64,149)	/
Total hospitalization cost^③^		1978 (1470.1,2452.4)	1470.3 (1010.5,1827.0)	2623.0 (1601.5,2814.0)	2207.0 (1993.5,2495.9)
Implant cost^③^		648.4 (298.9,990.9)	291.4 (176.3,438.0)	751.4 (620.5,784.9)	996.5 (954.6,1061.6)
Duration of cast immobilization^③^		33 (30,40)	33 (30,37)	35 (27,50)	34 (30,39)
Cubitus varus^①^					
	Yes	3 (5.9%)	1 (5%)	2 (14.3%)	0
	No	48 (94.1%)	19 (95%)	12 (85.7%)	17 (100%)
Spurring^①^	Yes	29 (56.9)	10 (50%)	9 (64.3%)	10 (58.8%)
	No	22 (43.1%)	10 (50%)	5 (35.7%)	7 (41.2%)
Elbow joint function^①^					
	Excellent	48 (94.1%)	19 (95.0%)	12 (85.7%)	17 (100%)
	Good	3 (5.9%)	1 (5.0%)	2 (14.3%)	0
	Fair	0	0	0	0
	poor	0	0	0	0

① n(%); ②Mean ± SD; ③Median (IQR)

Among the 51 patients who were followed up, there were no significant differences in gender (*p* = 0.347), age (*p* = 0.530), postoperative complications (*p* = 0.183, *p* = 0.696), postoperative plaster fixation time (*p* = 0.915), or postoperative elbow function evaluation (*p* = 0.237) among the different internal fixation material treatment groups (Groups A-C). However, there were significant differences in length of stay, surgical time, hospitalization costs, and internal fixation material costs (*p* < 0.001, *p* < 0.001, *p* < 0.001, *p* = 0.015, respectively; Table 3). The recovery process of each group is shown in Supplementary Figure.

**Table 3:** Comparison of treatment groups with different internal fixation materials

Variable	*P*
Gender	0.347
Age, years	0.530
Length of hospital stay	< 0.001
Operation time	< 0.001
Hollow screw versus Absorbable screw	1.000
Hollow screw versus Kirschner wire	0.002
Absorbable screw versus Kirschner wire	0.002
Time to implant removal	< 0.001
Kirschner wire versus Hollow screw	< 0.001
Total hospitalization cost	< 0.001
Kirschner wire vs. hollow screw	< 0.001
Kirschner wire versus absorbable screw	< 0.001
Hollow screw versus absorbable screw	1.000
Implant cost	< 0.001
Kirschner wire versus hollow screw	0.015
Kirschner wire versus absorbable screw	< 0.001
Hollow screw versus absorbable screw	0.005
Duration of cast immobilization	0.915
Carrying angle at final follow-up	0.367
Cubitus varus	0.183
Spurring	0.696
Mayo elbow performance score, *n* (%)	0.237
Satisfaction	0.602

## Discussion

Jakob III lateral condyle fracture in children is the most serious type of lateral condyle fracture. The current mainstream treatment is open reduction and Kirschner wire fixation, followed by plaster external fixation after surgery. However, metal hollow screw internal fixation treatment options also exist [17]. While there have been prior studies on the application of absorbable materials in orthopedics, there are relatively few studies on the application of absorbable materials in the treatment of pediatric elbow fractures [14]. In this study, we found that absorbable hollow screw internal fixation materials have the same efficacy as mainstream Kirschner wire materials and metal hollow screw materials in the treatment of Jakob III lateral condyle fractures in children in terms of postoperative complications and postoperative elbow function evaluation (*p* = 0.237). In addition, no differences in postoperative complications were found (*p* = 0.183, *p* = 0.696), suggesting that absorbable hollow screw materials can also achieve stable fixation in severe lateral condyle fractures in children. Moreover, owing to the absorption characteristics of absorbable materials, the trauma of removing internal fixation materials is avoided, which has many clinical advantages. Although there were no complications such as superficial infection of incision and early closure of epiphysis in this study, and it cannot fully explain that the probability of superficial infection in type III lateral humeral condylar fracture is very low, which may only be affected by the sample size.

Lal Sahu et al. noted that children with severe lateral humeral condylar fractures, such as those with type III fractures, direct open reduction and surgical treatment of this type of fracture have been widely recognized [18]. There are several differences in the choice of Kirschner wire insertion in clinical practice. Some studies [19] suggest that fixation with three Kirschner wires has a good supporting effect on provides sufficient stability, avoids complications such as displacement and cubitus varus deformity, and accelerates fracture healing. On the basis of two needles, a third crossed Kirschner wire is added to ensure multiplanar stability [20]. Currently, the common internal fixation materials for this type of fracture are the classical Kirschner wire internal fixation and the absorbable material internal fixation developed in recent years. Kirschner wires [21] are selected by surgeons because of their convenient fixation, minimal degree of trauma, simplicity and low cost. However, Kirschner wires have problems with elbow stiffness, nonunion, pin tract infection, and lateral protrusion. Cannulated metal screws can reduce the infection rate and improve the stability of the fracture block, but requires a second anesthetic procedure for implant removal, and the cannulated metal screws are at risk of damaging the epiphysis [22].

With the development of biomaterial science, absorbable materials, including bioabsorbable synthetic polyethylene glycol ester, polylactide and polydioxanone polymer devices [23], have no obvious immune response in vivo, and the degradation products (water and carbon dioxide) can be metabolized and excreted in vivo, which is increasingly widely used in orthopedic surgery and traumatology. At present, absorbable materials can also be applied for the treatment of supracondylar fractures of the humerus and other malformations in children [24]. Many studies on orthopedics and trauma have confirmed that these new bioabsorbable materials have good safety and efficacy and have no obvious adverse effects on the human body [25, 26]. The application of absorbable screws in the internal fixation of humeral lateral condyle fractures in children is simple and reliable. The fracture ends are closely combined, the clinical healing time of the fracture is shortened, and the curative effect is definite. It is a good internal fixation method [27]. Moreover, the elastic modulus of absorbable screws (about 3–5 MPa) is close to that of children’s bones, which can reduce the stress shielding caused by metal implants and promote bone remodeling. The most important thing is that absorbable screws can be absorbed by the body without the need for secondary surgery to remove the internal fixator, which greatly reduces the risk of reoperation and the possibility of surgical incision infection [28]. However, it should be emphasized that absorbable screws should be used carefully for young children. The smaller the metaphyseal fragment available for fixation, the more difficult the fixation is, and, ultimately, higher cost compared to Kirschner wires [29]. In this study, the material used in the absorbable cannulated screw fixation group (Group C) was B-ASC-35/40 absorbable cannulated screw produced by Bioretec, and its core component was poly-L-lactic acid (PLLA) and hydroxyapatite (HA) composite material. Although there were no adverse reactions in the absorbable screw group in this study, there were still risks of degradation-related local inflammatory reactions and mismatch between degradation rate and bone healing.

In terms of price, absorbable cannulated screws are more expensive than Kirschner wires and metal cannulated screws. This price difference is mainly due to their unique absorbable characteristics and complex manufacturing processes. In this study, the average total hospitalization cost of the Kirschner wire group was 1470.3$, which was the lowest among the three groups; the average total hospitalization cost of the cannulated screw group (including surgical fixation hospitalization and removal of cannulated screw hospitalization) was 2623.0$, which was the highest among the three groups. The average total hospitalization cost of the absorbable screw group was 2207.0$, between the two. However, Kirschner wires are typically removed in an outpatient setting, and this study did not include the cost of taking the Kirschner wire in the outpatient department. At the same time, considering that absorbable materials can avoid the psychological pressure caused by secondary surgery, reduce the family’s economic burden and reduce physical trauma, their cost-effectiveness is gradually increasing.

Clinically, for displaced lateral condyle fractures, the use of Kirschner wire fixation is more commonly used, but some recent studies have advocated the use of screw fixation. For example, the study included a randomized controlled study and three retrospective cohort studies involving 240 patients (Kirschner wire: 118 cases, screw: 122 cases). There was no difference in the clinical effect evaluated according to the Hardcree criteria between the two groups (*P* = 0.54) [1]. Wen Chao Li compared Kirschner wire and AO type hollow screw internal fixation in the treatment of displaced lateral condylar fracture of humerus, and concluded that there was no significant difference in the clinical treatment effect between the two groups [13]. Li’s study included 85 patients and pointed out that both Kirschner wire and absorbable material were safe and effective in the treatment of children’s lateral condyle fractures [30].

In recent years, absorbable screws have been widely used in trauma medicine. In addition to elbow fractures, they are also used in ankle fractures and anterior cruciate ligament reconstruction [31, 32]. There is no difference in clinical effects between absorbable screws and traditional Kirschner wires and metal screws. Absorbable screw is preferred due to its advantages of safety, cleanliness and avoiding the removal procedure associated with metallic implants.

In this study, the selection of fixation material (Kirschner wire, metal hollow screw, absorbable hollow screw) was based on the following types of criteria: objective characteristics of the fracture itself: this is the most important decision-making factor. All choices are based on preoperative X-ray imaging evaluation: the fracture type is Jakob type III lateral condyle fracture, displacement > 2 mm and flipping. Secondly, the choice of fixation adhered to the principles outlined in the 2022 edition of the “Expert Consensus on the Diagnosis and Treatment of Humeral Lateral Condylar Fractures in Children,” which is not the subjective choice of surgeons. Finally, weigh the fixed strength and postoperative activity needs combined with the family’s expectations of the postoperative recovery process, the decision is made jointly by the doctor and the parents.

In the selection of internal fixation materials, we also need to consider the harm caused by multiple operations to the children’s body and the negative effects of anesthesia. These factors cannot be ignored. The perioperative non-specific adaptation response is a pattern of physiological and pathophysiological changes that occur after surgical trauma. It is affected by the size, invasiveness and duration of the operation. This adaptation response can sometimes be detrimental and can produce systemic inflammatory response syndrome (SIRS), characterized by hypermetabolism and hyper-catabolism [33].

Robert Ivascu et al. noted anesthetics and analgesics used throughout the perioperative period may regulate the innate immune system, adaptive immune system and inflammatory system, and may cause the immune system of children to be at a low level in the short term [34]. Therefore, the damage caused by secondary or even multiple surgeries and the negative effects of anesthesia are also important factors that surgeons should consider when choosing internal fixation materials for treatment. Therefore, in the case of equal surgical efficacy, secondary surgery should be avoided as much as possible. If one operation can solve the problem, it is the best.

As a retrospective investigation, A limitation of this study is its retrospective, non-randomized design. we explicitly highlight this limitation due to its notable influence on the strength of causal inference.

As a retrospective study, probabilistic grouping through randomization was not feasible. In each group, the selection of internal fixation materials is dictated by the actual decisions made during clinical practice rather than by a random process predefined in the research design. This situation may give rise to uncontrolled confounding factors among the groups. In clinical practice, the random allocation of clearly effective therapies for type III lateral condylar fractures may involve ethical disputes. As noted in the literature, observational studies remain necessary in many clinical scenarios [35]. This conclusion is still an exploratory discovery rather than definitive evidence. According to the GRADE framework for evaluating evidence, the lack of random allocation will reduce the quality level of evidence, so the conclusion of this study needs to be verified by subsequent RCTs. In summary, we acknowledge that the lack of randomization limits the strength of our conclusions, and have tried to mitigate its impact through transparent methodological reports. The value of this study is more reflected in the selection of internal fixation materials for the treatment of type III lateral condylar fractures, rather than the establishment of final clinical practice guidelines.

## Conclusion

For patients with severe humeral condylar fractures, different internal fixation materials can achieve good functional recovery. The absorbable hollow screw internal fixation material is a more feasible fixation material option for children with humeral condylar fractures because of its absorbable properties and its ability to avoid the trauma of internal fixation removal.

## Supplementary Information

Below is the link to the electronic supplementary material.

Supplementary Material 1

## Data Availability

No datasets were generated or analysed during the current study.

## References

[1] Cho YJ, Kang SH, Kang MH. K-wire versus screws in the fixation of lateral condyle fracture of humerus in pediatrics: a systematic review and meta-analysis. BMC Musculoskelet Disord. 2023;24(1):649. 10.1186/s12891-023-06780-5. 37573303 10.1186/s12891-023-06780-5 PMC10423410

[2] Milch H. Fractures and fracture dislocations of the humeral condyles. J Trauma. 1964;4:592–607. 10.1097/00005373-196409000-00004. 14208785 10.1097/00005373-196409000-00004

[3] Jakob R, Fowles JV, Rang M, Kassab MT. Observations concerning fractures of the lateral humeral condyle in children. J Bone Joint Surg Br. 1975;57(4):430–6. 1104630

[4] Abzug JM, Dua K, Kozin SH, Herman MJ. Current concepts in the treatment of lateral condyle fractures in children. J Am Acad Orthop Surg. 2020;28(1):e9–19. 10.5435/jaaos-d-17-00815. 31268870 10.5435/JAAOS-D-17-00815

[5] Aibara N, Takagi T, Seki A. Late displacement after lateral condylar fractures of the humerus. J Shoulder Elbow Surg. 2022;31(10):2164–8. 10.1016/j.jse.2022.06.003. 35926831 10.1016/j.jse.2022.06.003

[6] Flynn JC. Nonunion of slightly displaced fractures of the lateral humeral condyle in children: an update. J Pediatr Orthop. 1989;9(6):691–6. 10.1097/01241398-198911000-00012. 2600178 10.1097/01241398-198911000-00012

[7] Ganeshalingam R, Donnan A, Evans O, Hoq M, Camp M, Donnan L. Lateral condylar fractures of the humerus in children: does the type of fixation matter? Bone Joint J. 2018;100-b(3):387–95. 10.1302/0301-620x.100b3.Bjj-2017-0814.R1. 29589493 10.1302/0301-620X.100B3.BJJ-2017-0814.R1

[8] Greenhill DA, Funk S, Elliott M, Jo CH, Ramo BA. Minimally displaced humeral lateral condyle fractures: immobilize or operate when stability is unclear? J Pediatr Orthop. 2019;39(5):e349–54. 10.1097/bpo.0000000000001311. 30531548 10.1097/BPO.0000000000001311

[9] Kassai T, Krupa Z, Józsa G, Hanna D, Varga M. Comparison of biodegradable and metallic tension-band fixation for paediatric lateral condyle fracture of the elbow. Injury. 2024;55(3):111403. 10.1016/j.injury.2024.111403. 39300617 10.1016/j.injury.2024.111403

[10] Koh KH, Seo SW, Kim KM, Shim JS. Clinical and radiographic results of lateral condylar fracture of distal humerus in children. J Pediatr Orthop. 2010;30(5):425–9. 10.1097/BPO.0b013e3181df1578. 20574257 10.1097/BPO.0b013e3181df1578

[11] Tan SHS, Dartnell J, Lim AKS, Hui JH. Paediatric lateral condyle fractures: a systematic review. Arch Orthop Trauma Surg. 2018;138(6):809–17. 10.1007/s00402-018-2920-2. 29574555 10.1007/s00402-018-2920-2

[12] Li WC, Xu RJ. Comparison of Kirschner wires and AO cannulated screw internal fixation for displaced lateral humeral condyle fracture in children. Int Orthop. 2012;36(6):1261–6. 10.1007/s00264-011-1452-y. 22179811 10.1007/s00264-011-1452-y PMC3353087

[13] Sinha S, Kumar A, Meena S, Jameel J, Qureshi OA, Kumar S. K wires or cannulated screws for fixation of lateral condyle fractures in children: a systematic review of comparative studies. Indian J Orthop. 2023;57(6):789–99. 10.1007/s43465-023-00873-y. 37214369 10.1007/s43465-023-00873-y PMC10192480

[14] Rokkanen PU, Böstman O, Hirvensalo E, Mäkelä EA, Partio EK, Pätiälä H, et al. Bioabsorbable fixation in orthopaedic surgery and traumatology. Biomaterials. 2000;21(24):2607–13. 10.1016/s0142-9612(00)00128-9. 11071610 10.1016/s0142-9612(00)00128-9

[15] Pribaz JR, Bernthal NM, Wong TC, Silva M. Lateral spurring (overgrowth) after pediatric lateral condyle fractures. J Pediatr Orthop. 2012;32(5):456–60. 10.1097/BPO.0b013e318259ff63. 22706459 10.1097/BPO.0b013e318259ff63

[16] Baessler A, Eason RR, Joyce MR, Dibaba DT, Wan JY, Azar FM, et al. Reliability testing of the mayo elbow performance score in post-operative patients. J Surg Orthop Adv. 2022;31(4):229–32. 36594979

[17] Zhu S, Zheng Y, Jiang Y, Yin H, Zhu D. Open versus closed reduction internal fixation for lateral condyle humeral fractures in children: a systematic review and meta-analysis. J Orthop Surg Res. 2023;18(1):322. 10.1186/s13018-023-03808-3. 37098573 10.1186/s13018-023-03808-3 PMC10131320

[18] Lal Sahu R. Percutaneous K wire fixation in pediatric lateral condylar fractures of humerus: a prospective study. Rev Esp Cir Ortop Traumatol (Engl Ed). 2018;62(1):1–7. 10.1016/j.recot.2017.10.005. 29157991 10.1016/j.recot.2017.10.005

[19] Bilgili F, Demirel M, Birişik F, Balcı H, Sunbuloglu E, Bozdag E. A new configuration of lateral-pin fixation for pediatric supracondylar humeral fracture: a biomechanical analysis. Acta Orthop Traumatol Turc. 2024;58(2):110–5. 10.5152/j.aott.2024.21091. 38705973 10.5152/j.aott.2024.21091 PMC11181288

[20] Jeon S, Ahn W, Oh J, Chung J, Choi J, Lee S. Finite element analyses of lateral condyle fracture fixation in paediatrics regarding configuration of Kirschner-wire. BMC Musculoskelet Disord. 2022;23(1):940. 10.1186/s12891-022-05897-3. 36307784 10.1186/s12891-022-05897-3 PMC9615206

[21] Schlitz RS, Schwertz JM, Eberhardt AW, Gilbert SR. Biomechanical analysis of screws versus K-wires for lateral humeral condyle fractures. J Pediatr Orthop. 2015;35(8):e93–7. 10.1097/bpo.0000000000000450. 25985374 10.1097/BPO.0000000000000450

[22] Lebel E, Weinberg E, Berenstein-Weyel TM, Bromiker R. Early application of the Ponseti casting technique for clubfoot correction in sick infants at the neonatal intensive care unit. J Pediatr Orthop B. 2017;26(2):108–11. 10.1097/bpb.0000000000000363. 28118300 10.1097/BPB.0000000000000363

[23] Rokkanen P, Böstman O, Vainionpää S, Vihtonen K, Törmälä P, Laiho J, et al. Biodegradable implants in fracture fixation: early results of treatment of fractures of the ankle. Lancet. 1985;1(8443):1422–4. 10.1016/s0140-6736(85)91847-1. 2861365 10.1016/s0140-6736(85)91847-1

[24] Li J, Rai S, Ze R, Tang X, Liu R, Hong P. Is bioabsorbable screw an alternative choice for displaced medial epicondylar fractures in adolescents: a comparative study of metallic cannulated lag screw versus bioabsorbable screw. Medicine (Baltimore). 2020;99(35):e22001. 10.1097/md.0000000000022001. 32871954 10.1097/MD.0000000000022001 PMC7458265

[25] Andrey V, Tercier S, Vauclair F, Bregou-Bourgeois A, Lutz N, Zambelli PY. Lateral condyle fracture of the humerus in children treated with bioabsorbable materials. Sci World J. 2013;2013:869418. 10.1155/2013/869418. 10.1155/2013/869418 PMC3817636 24228016

[26] Takada N, Otsuka T, Suzuki H, Yamada K. Pediatric displaced fractures of the lateral condyle of the humerus treated using high strength, bioactive, bioresorbable F-u-HA/PLLA pins: a case report of 8 patients with at least 3 years of follow-up. J Orthop Trauma. 2013;27(5):281–4. 10.1097/BOT.0b013e318269ba8e. 22832435 10.1097/BOT.0b013e318269ba8e

[27] Su Y, Chen K, Qin J. Retrospective study of open reduction and internal fixation of lateral humeral condyle fractures with absorbable screws and absorbable sutures in children. Medicine (Baltimore). 2019;98(44):e17850. 10.1097/md.0000000000017850. 31689876 10.1097/MD.0000000000017850 PMC6946392

[28] Warwick D. Intermittent pneumatic compression prophylaxis for proximal deep venous thrombosis after total hip replacement. J Bone Joint Surg Am. 1998;80(1):141–2. 9469318

[29] Woolf SK, Gross RH. Perceptions of allograft safety and efficacy among spinal deformity surgeons. J Pediatr Orthop. 2001;21(6):767–71. 11675552

[30] Li J, Rai S, Liu Y, Ze R, Tang X, Liu R, et al. Is biodegradable pin a good choice for lateral condylar fracture of humerus in children: a comparative study of biodegradable pin and Kirschner wire. Medicine (Baltimore). 2020;99(33):e21696. 10.1097/md.0000000000021696. 32872043 10.1097/MD.0000000000021696 PMC7437816

[31] Laupattarakasem P, Laopaiboon M, Kosuwon W, Laupattarakasem W. Meta-analysis comparing bioabsorbable versus metal interference screw for adverse and clinical outcomes in anterior cruciate ligament reconstruction. Knee Surg Sports Traumatol Arthrosc. 2014;22(1):142–53. 10.1007/s00167-012-2340-8. 23238925 10.1007/s00167-012-2340-8

[32] Tang J, Hu JF, Guo WC, Yu L, Zhao SH. Research and application of absorbable screw in orthopedics: a clinical review comparing PDLLA screw with metal screw in patients with simple medial malleolus fracture. Chin J Traumatol. 2013;16(1):27–30. 23384867

[33] Cusack B, Buggy DJ. Anaesthesia, analgesia, and the surgical stress response. BJA Educ. 2020;20(9):321–8. 10.1016/j.bjae.2020.04.006. 33456967 10.1016/j.bjae.2020.04.006 PMC7807970

[34] Ivascu R, Torsin LI, Hostiuc L, Nitipir C, Corneci D, Dutu M. The surgical stress response and anesthesia: a narrative review. J Clin Med. 2024. 10.3390/jcm13103017. 38792558 10.3390/jcm13103017 PMC11121777

[35] Power MC, Engelman BC, Wei J, Glymour MM. Closing the gap between observational research and randomized controlled trials for prevention of Alzheimer disease and dementia. Epidemiol Rev. 2022;44(1):17–28. 10.1093/epirev/mxac002. 35442427 10.1093/epirev/mxac002 PMC10362937

