# Supplementary material for: The effect of different internal fixation materials on Jakob type III lateral humeral condyle fractures in pediatric patients
Source: J Orthop Surg Res. 2025 Dec 2;21:14. doi: 10.1186/s13018-025-06547-9 (PMC12777173; doi:10.1186/s13018-025-06547-9)
Supplement: Supplementary file 1 — Supplementary Material 1 [file 13018_2025_6547_MOESM1_ESM.docx]

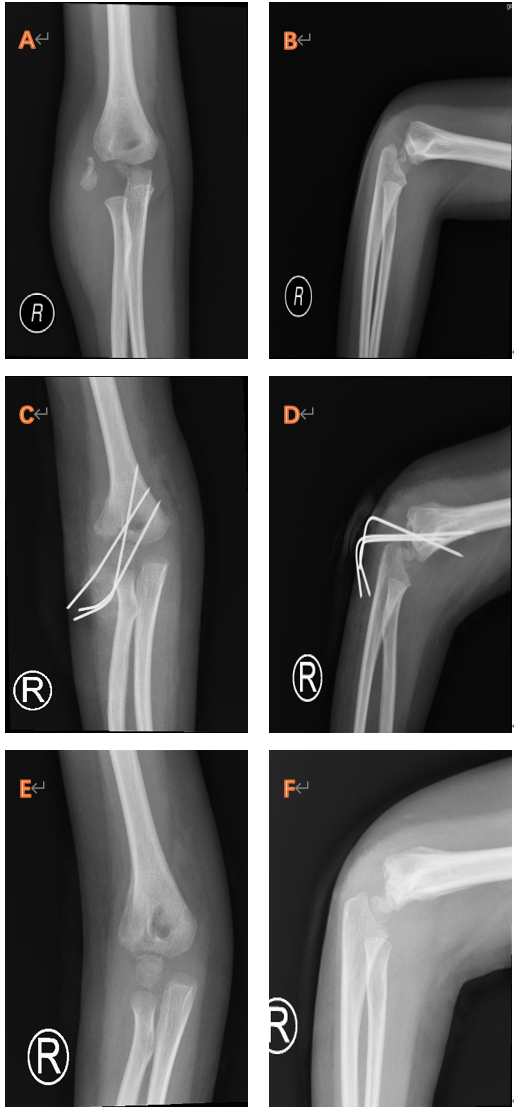


Figure 1 In the Kirschner wire group, the X-ray positive and lateral positions were taken before operation（A/B）, 1 month after operation （C/D）and after taking Kirschner wire（E/F）.


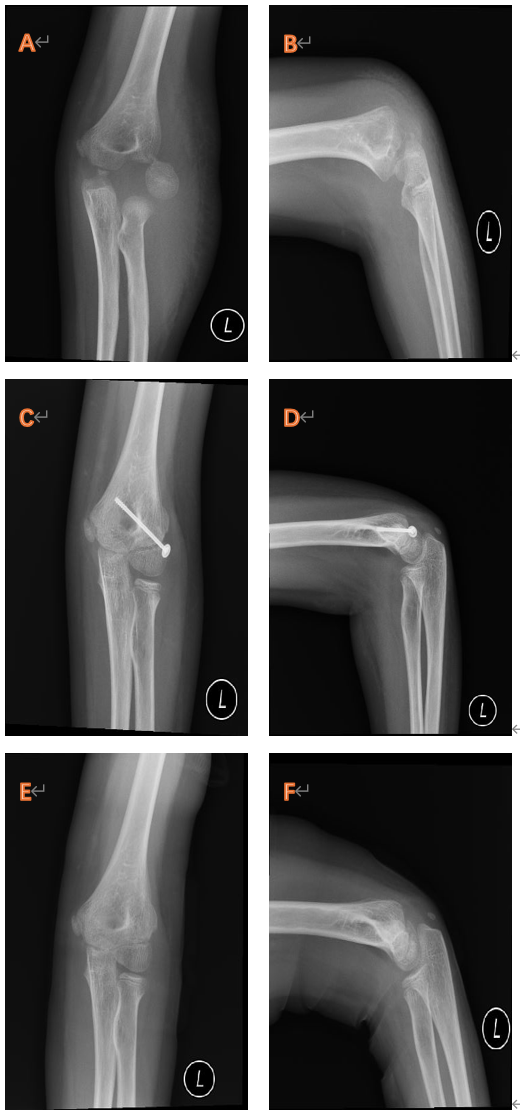


Figure 2 In the cannulated screw group, the X-ray was positive and lateral before operation（A/B）, 1 month after operation(C/D) and after screw removal(E/F).


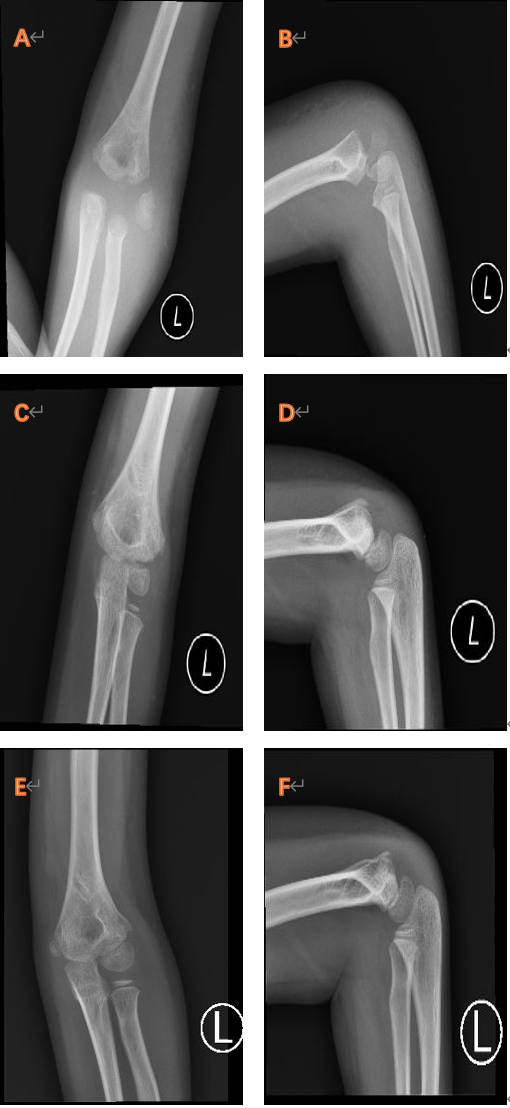


Figure 3 In the absorbable screw group, the X-ray was positive and lateral before operation（A/B）, 1 month after operation(C/D) and at the last follow-up(E/F).
